# Modular Neural Networks with Fully Convolutional Networks for Typhoon-Induced Short-Term Rainfall Predictions

**DOI:** 10.3390/s21124200

**Published:** 2021-06-18

**Authors:** Chih-Chiang Wei, Tzu-Heng Huang

**Affiliations:** Department of Marine Environmental Informatics and Center of Excellence for Ocean Engineering, National Taiwan Ocean University, Keelung 20224, Taiwan; Azeros@proguide.com.tw

**Keywords:** typhoon, rainfall, convolutional networks, image segmentation, prediction

## Abstract

Taiwan is located at the edge of the northwestern Pacific Ocean and within a typhoon zone. After typhoons are generated, strong winds and heavy rains come to Taiwan and cause major natural disasters. This study employed fully convolutional networks (FCNs) to establish a forecast model for predicting the hourly rainfall data during the arrival of a typhoon. An FCN is an advanced technology that can be used to perform the deep learning of image recognition through semantic segmentation. FCNs deepen the neural net layers and perform upsampling on the feature map of the final convolution layer. This process enables FCN models to restore the size of the output results to that of the raw input image. In this manner, the classification of each raw pixel becomes feasible. The study data were radar echo images and ground station rainfall information for typhoon periods during 2013–2019 in southern Taiwan. Two model cases were designed. The ground rainfall image-based FCN (GRI_FCN) involved the use of the ground rain images to directly forecast the ground rainfall. The GRI combined with rain retrieval image-based modular convolutional neural network (GRI-RRI_MCNN) involved the use of radar echo images to determine the ground rainfall before the prediction of future ground rainfall. Moreover, the RMMLP, a conventional multilayer perceptron neural network, was used to a benchmark model. Forecast horizons varying from 1 to 6 h were evaluated. The results revealed that the GRI-RRI_MCNN model enabled a complete understanding of the future rainfall variation in southern Taiwan during typhoons and effectively improved the accuracy of rainfall forecasting during typhoons.

## 1. Introduction

Taiwan is located in the northwestern Pacific Ocean within an area frequently hit by typhoons. After their formation, typhoons often move along the west Pacific Ocean and strike Taiwan with strong winds and torrential rain. On average, three to four typhoons land in Taiwan each year [1]. Southern Taiwan lies in a subtropical zone. The main rainy season in southern Taiwan is the typhoon season between May and October. Nearly no rainfall occurs in the other months. Therefore, the main water source in southern Taiwan is the rainfall caused by typhoons. However, the short-duration heavy rainfall of typhoons not only provides abundant water but also causes disasters, such as debris flows, river water surges, and downstream flooding [2,3]. Typhoons commonly strike southern Taiwan, for example Typhoon Fung-Wong in 2014 and Typhoons Nepartak, Meranti, and Typhoon Megi in 2016, which caused severe disasters and property losses [4,5]. Therefore, an accurate rainfall forecasting model is urgently required for southern Taiwan to accurately predict the real-time rainfall during typhoon periods and prevent the disasters resulting from heavy rainfall in local areas.

In recent years, considerable developments have occurred in machine learning (ML). Scholars have used various ML-based algorithms along with ground observation data, namely one-dimensional (1-D) data, for precipitation estimation and prediction; for example, artificial neural networks [6,7,8,9,10] and support vector machines [11,12] have been employed to predict rainfall using 1-D ground rainfall data. Although rain gauges provide relatively accurate point rainfall estimates near the ground surface, they cannot effectively capture the spatial variability of rainfall [13,14].

Remote sensing has attracted increasing attention in weather analysis and forecasting. Various types of image data have been collected for remote sensing applications. The development of weather surveillance radars has enabled quantitative precipitation estimation with extremely high spatial resolutions. Weather radars, which have the advantages of wide coverage and round-the-clock observation, are critical devices for meteorological observation [15]. Accordingly, the application of two-dimensional (2-D) radar images compensates for the insufficient 1-D spatial rainfall data collected from land-based observation stations. Many studies have used the statistical relationships between the radar reflectivity and the rain rate or nonlinear regression to establish rainfall estimation models. These studies have achieved favorable outcomes [16,17,18,19,20,21,22,23,24,25]. However, the interpretation of these image data is a crucial emerging topic.

Deep learning (DL) is a prominent branch of ML. DL mainly involves using neural-network-based ML algorithms to develop advanced computational technology that can be applied in image recognition. A DL neural network structure is a multilayer neural network architecture that uses two-dimensional matrices to calculate images. Therefore, advanced computer processing units (i.e., graphics cards) are required to execute DL tasks successfully [26]. The convolutional neural networks (CNNs) developed by LeCun et al. [27] is a basic DL image recognition technology. The structure of the CNN model comprises a convolutional layer and pooling layer. A complete CNN model is established using a fully connected layer, which converts two-dimensional images into one-dimensional arrays, and multilayer perceptron network model structures [28]. Such a network structure enables the CNN model to achieve favorable image recognition accuracy [29,30,31,32]. CNN algorithms have also been successfully applied to rainfall estimation and hydrological problems. For example, Pan et al. [33] used CNN model stacks with several convolution and pooling operators to extract intricate and valuable circulation features for precipitation estimation. Sadeghi et al. [34] estimated the precipitation rate by processing images in the infrared (IR) and water vapor bands (obtained from geostationary satellites) by using CNNs. Wang et al. [35] proposed the dilated causal CNN model to predict the water level changes during typhoons. Wei [36] proposed a regional extreme precipitation and construction suspension estimation system and used a deep CNN model to enhance the extreme rainfall forecasting capability of this system. The aforementioned studies developed CNNs for precipitation susceptibility mapping by using various 2-D remote images.

Newly emerging DL skills were employed in the study case. First, a fully convolutional network (FCN) developed by Long et al. [37] was employed to conduct image recognition. The FCN was developed as an extension of the CNN for semantic segmentation to address the shortcomings of CNN and increase the prediction accuracy for the rapid recognition of various object representations. To facilitate the pixel classification of images, upsampling was conducted in the FCN model for classifying every pixel on the feature map of the final convolutional layer. The FCN model used deconvolution to match the class of every pixel in a feature map with the corresponding class in the original image and thus solved the problem of semantic segmentation. To the best of our knowledge, few studies have used FCNs for rainfall estimation and prediction. Moreover, Eppel [38] proposed modular convolutional neural networks (MCNNs) that apply FCNs to segment an image into vessel and background area; in that study, the vessel region was used as an input for a second net that recognized the contents of a glass vessel.

The current study developed a DL-based rainfall prediction model, for which the source data are both 1-D ground observation data and 2-D remote sensing imageries, to predict precipitation during typhoons. Southern Taiwan was selected as the research area. This study used the hourly rainfall data of ground stations and radar echo images in southern Taiwan to establish an hourly rainfall forecast model. Toward the aforementioned goal, this study has the following features:

(1) This study employed an FCN, which employs the convolutional and pooling layers for extracting image features, to predict the precipitation during typhoons.
(2) To address the input–output patterns in the FCN modeling process using 2-D array data, this study converted the rainfall data of ground stations into 2-D images.
(3) This study employed the net architecture of MCNN with FCNs, which enabled the integration of the radar echo image and ground observation data as model inputs for enhancing the accuracy of rainfall intensity prediction.

## 2. Experimental Area and Data

### 2.1. Region and Gauges

**Figure 1:**
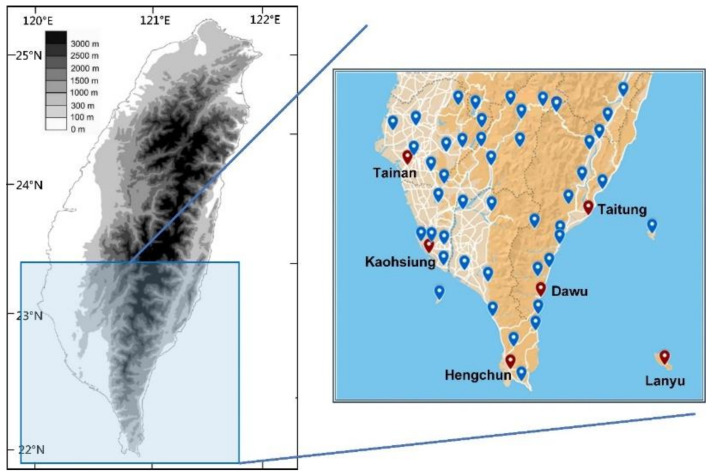
Map of the research area.

The longitude and latitude ranges of southern Taiwan are 120.11–121.59° E and 22.00–23.34° N, respectively (Figure 1). The area of southern Taiwan is 11,434 km^2^, which accounts for 31.59% of the total area of Taiwan. As displayed in the right part of Figure 1, southern Taiwan has 51 weather stations, comprising six Central Weather Bureau (CWB) weather stations (red dots) and 45 automatic detection stations (blue dots). The CWB weather stations are located at Tainan, Kaohsiung, Hengchun, Taitung, Dawu, and Lanyu (coordinates are provided in Table 1). This study used the six CWB weather stations as the experimental sites.

The left part of Figure 1 indicates that the Central Mountain Range (CMR) runs south–north and divides Taiwan into the eastern and western regions. The total length of the CMR is approximately 340 km, and its width from east to west is approximately 80 km. The average altitude of the range is approximately 2500 m [39]. The Tainan and Kaohsiung stations are located to the west of the CMR, the Hengchun station is located to the south of the CMR, the Taitung and Dawu stations are located to the east of the CMR, and the Lanyu station is located in an outlying island (bottom right of Figure 1).

### 2.2. Typhoons and Radar Mosaics

In Taiwan, the CWB creates radar echo images (REIs) by using different colors to represent the spatial echo intensity of the reflected signals received by radars from rain particles [40]. REIs are used to reflect the variations of water vapor during typhoon circulation. Wu and Kuo [41] indicated that useful typhoon-related data can be obtained when a typhoon affects Taiwan by setting up an around-the-island Doppler radar network, enhanced surface rain gauge network, and integrated sounding system. This study collected radar images starting from 2013 because the resolution and color appearance of these images were different from those of the radar images captured before 2013. According to the CWB’s Typhoon Database [42], 22 typhoon events occurred in Taiwan from 2013 to 2019 (Table 2).

According to the CWB, the maximum wind speeds of mild, moderate, and severe typhoons are 17.2–32.6, 32.7–50.9, and >51 m/s, respectively. Seven severe, six moderate, and nine mild typhoons occurred in southern Taiwan during the study period.

**Figure 2:**
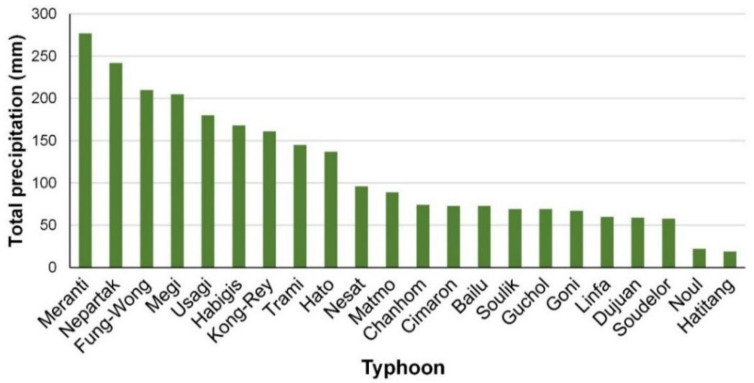
Total precipitation of typhoons between 2013 and 2019.

Figure 2 displays the accumulated precipitation of each typhoon in descending order. The top nine typhoons in terms of precipitation, namely Typhoons Trami, Kong-Rey, Usagi, Habigis, Fung-Wong, Nepartak, Meranti, Megi, and Hato, had relatively high precipitation (accumulated precipitation > 100 mm), whereas the others had relatively low precipitation.

**Figure 3:**
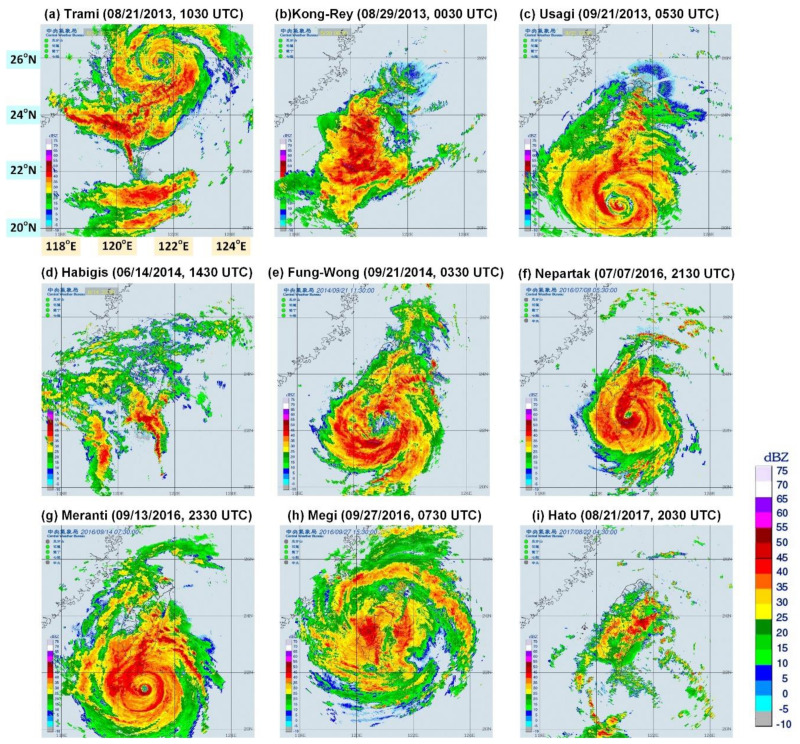
Collected original radar echo images: (**a**) Typhoons Trami, (**b**) Kong-Rey, (**c**) Usagi, (**d**) Habigis, (**e**) Fung-Wong, (**f**) Nepartak, (**g**) Meranti, (**h**) Megi, and (**i**) Hato (the size of each map is 1024 × 1024 pixels) (The radar mosaic images were produced by the Central Weather Bureau [42]).

This study collected 1412 radar mosaic images with a resolution of 1024 × 1024 pixels. Here, one pixel corresponded to an actual distance of 0.7 × 0.7 km. Figure 3 displays the REIs of nine typhoons approaching the study region. These typhoons all resulted in accumulated precipitation >100 mm.

## 3. Model Development

This study used the Python programming language to establish models. The Tensorflow (version 2.1) and Keras libraries of Python were used for ML computation. The model computation environment was an ASUS-TS300E9 computer (ASUSTek Computer Inc., Taipei City, Taiwan). The computer clock rate was 3.5 GHz. The computer included 16 GB RAM (DDR4-2400) and a GeForce GTX 1080 Ti X 11G graphics card (Micro-Star International Co., Ltd., New Taipei City, Taiwan).

### 3.1. Data Division

This study divided the data of typhoon events into training, validation, and testing sets. The training sets were used to tune the model parameters, and the validation sets were used to verify the trained model. To avoid the data leakage and bias problem in the rainfall prediction model, this study randomly split the typhoons ranked 1 to 9 in terms of precipitation into training, validation, and testing sets; that is, rank = 2, 6 and 9 for training set (Nepartak, Habigis, and Hato), rank = 3, 5 and 8 for validation set (Fung-Wong, Usagi, and Trami), and rank = 1, 4 and 7 for testing set (Meranti, Megi, and Kong-Rey). In addition, the remaining typhoons (relative low precipitation) were added for training set. In total, the training, validation, and testing sets comprised 926, 240 and 246 hourly records, respectively.

### 3.2. Image Preprocessing

In the study, all the inputs and outputs in the modeling process in this study were two-dimensional images. First, when labeling the REI images, the latitudinal and longitudinal range of the original radar images was 117.32–124.79° E and 21.70–27.17° N (Figure 3). Because the original images had a wide geographical range, cropping was required to obtain the image size of study area (120.11–121.59° E and 22.00–23.34° N). Therefore, the raw REIs were cut to a size of 192 × 192 pixels to completely cover the study area. According to the legend of dBZ (Figure 3), there are 17 colors (where dBZ ranging from −10 to 75 dBZ, divided by 5 dBZ). Therefore, the number of categories was 17. These REI images were then encoded into RGB channels (i.e., red, green, and blue) and pixel values at each channel are integer values between 0 and 255. Here, a one-hot encoding was applied to the RGB representation of an REI image when pixel-based images were used as the model inputs.

Second, the rainfall data of ground stations had to be converted into two-dimensional ground rainfall images (namely GRIs). The inverse distance weighting method proposed by Shepard [43] was employed. In this method, an interpolating function is used to identify an interpolated value at a given point based on samples by using the inverse distance weighting method as follows:

(1) $$u\left(x\right)=\left\{\begin{array}{c} \frac{\sum_{i=1}^{N}w_{i}\left(x\right)u_{i}}{\sum_{i=1}^{N}w_{i}\left(x\right)},\;\mathrm{if}\;d\left(x,x_{i}\right)\neq 0\;\mathrm{for}\;\mathrm{all}\;i\\ u_{i},\;\mathrm{if}\;d\left(x,x_{i}\right)=0\;\mathrm{for}\;\mathrm{some}\;i \end{array}\right.$$

where $w_{i}\left(x\right)=\frac{1}{d{\left(x,x_{i}\right)}^{p}}$ is a weighting function; **x** denotes an interpolated (unknown) point; **x***_i_* is an interpolating (known) point; *d* is a given distance from **x***_i_* to **x**; *N* is the total number of known points used in interpolation; and *p* is a positive real number, called the power parameter.

**Figure 4:**
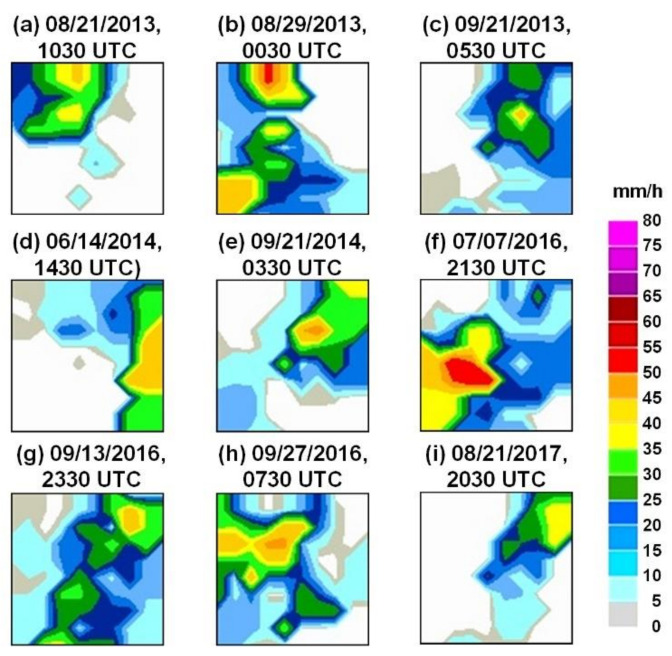
Generated GRIs of Typhoons (**a**) Trami, (**b**) Kong-Rey, (**c**) Usagi, (**d**) Habigis, (**e**) Fung-Wong, (**f**) Nepartak, (**g**) Meranti, (**h**) Megi, and (**i**) Hato. (the size of each map is 192 × 192 pixels).

This study employed the commonly used *p* = 2 and subsequently identified the suitable *N* value. This study found that when *N* ≤ 4, the GRIs were varied; however, when *N* ≥ 5, the GRIs were more stable and invariant. Figure 4 depicts the GRIs of Typhoons Trami, Kong-Rey, Usagi, Habigis, Fung-Wong, Nepartak, Meranti, Megi, and Hato using the inverse distance weighting method when *p* = 2 and *N* = 5. Here, the size of GRI maps is the same as the cropped REI maps (i.e., 192 × 192 pixels). Subsequently, when labeling the GRI images, this study partitioned the precipitation scale into several intervals to label the categorical values. According to the collected typhoons, the range of rain rate from 0 to 76 mm/h. This study divides the rain rate by 5 mm/h. Here, we let the no rain as a special case, as class “0”. Thus, the total number of rain intensity categories was 17. For example, if the rain rate was 13 mm/h, it was labeled as class “3”. Then, each pixel of the GRI images can be labeled by classes 0 to 16. Finally, these GRI images were encoded into RGB channels when the GRI images were used as the model targets.

### 3.3. Designed Model Cases

**Figure 5:**
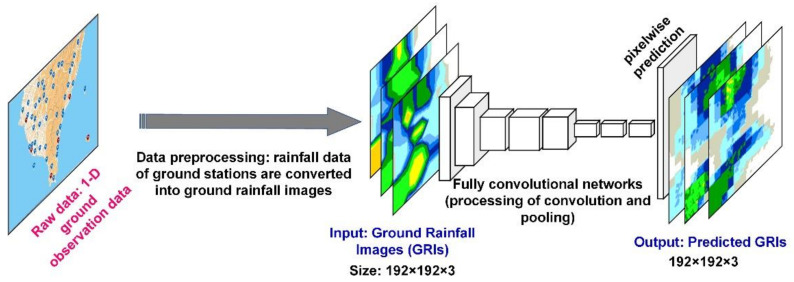
Architecture of the GRI-based fully convolutional networks. (an image of GRI contains a three-dimensional array of size *h* × *w* × *d*, where *h* = 192 and *w* = 192 are spatial dimensions, and *d* = 3 is the color channel dimension).

In this study, two rainfall prediction models were developed on the basis of two types of neural networks: The GRI-based FCNs (GRI_FCNs) and GRI combined with rain retrieval image (RRI)-based MCNNs (GRI-RRI_MCNNs). The developed GRI_FCN (Figure 5) adopted segmentation steps using a standard FCN, which segmented the image into objects by classifying every pixel in the image into one of a given set of categories. The framework of the GRI_FCN included input, downsampling, upsampling, and output layers. Before FCN modeling was conducted, the 1-D rainfall data of ground stations were converted into 2-D GRIs. In the GRI_FCN model, the GRIs were adopted to predict the ground rainfall directly, and the output results were the predicted GRIs.

**Figure 6:**
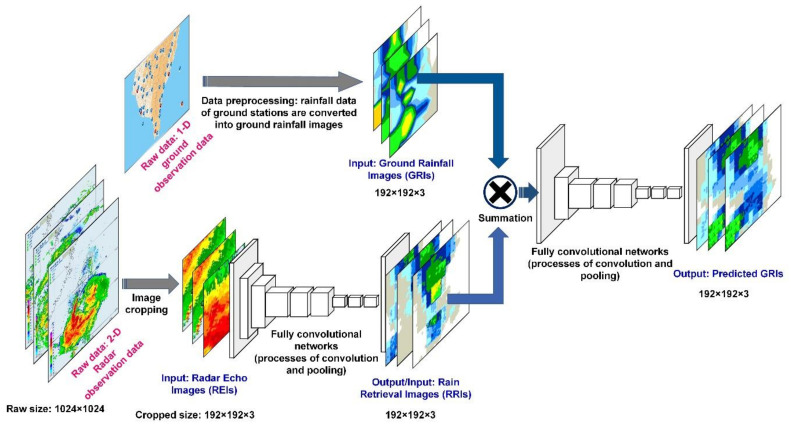
Architecture of blending GRI-RRI-based modular convolutional neural networks. (the images of GRI, REI and RRI contain a three-dimensional array of size 192 × 192 × 3).

The GRI-RRI_MCNN model employed a modular semantic segmentation approach using serially connected FCN networks. The first FCN net identified current ground precipitation, and the output of this net (i.e., rain retrievals) was used by a second FCN net to identify and segment the future ground precipitation (i.e., rain predictions; Figure 6). The GRI-RRI_MCNN involved two steps: in step 1, REIs were used to retrieve the ground rainfall (the GRIs are the model learning targets). The outputs were RRIs. Step 2 involved the fusion (using a summation method) of the RRIs and GRIs obtained in step 1 to create new images. These new images were subsequently used as the input to predict the ground rainfall, and the output results were the predicted GRI images.

The convolution and pooling processes of the FCN in GRI_FCNs and GRI-RRI_MCNNs were identical to those of the CNN. The net architecture of the CNN has been described by [27,44]. In general, CNNs are constructed by stacking two types of interweaved layers: convolutional and pooling (subsampling) layers [45]. The convolutional layer is the core component of a CNN. This layer outputs feature maps by computing the dot product between the local region in the input feature maps and a filter. The pooling layer performs downsampling on feature maps by computing the maximum or average value of a subregion [46]. An FCN has more neural net layers than a CNN does. An FCN conducts upsampling on the feature map of the final convolution layer. This design enables FCN models to restore the size of the output results to that of the raw input images. Therefore, the classification is performed for every raw image pixel [37]. An FCN can theoretically accept an input image of any size and produce output images of the same size because an FCN is trained end-to-end for pixel-to-pixel semantic segmentation (or pixel-wise prediction).

When running the GRI_FCN and GRI-RRI_MCNN models, the parameter settings of the convolutional and pooling layers were as follows: kernel size = (2, 2), padding method = same, maxpooling with filter size = (2, 2), strides = (2, 2), and the activation function = rectified linear unit function. Moreover, the settings of output layers were as follows: kernel size = (8, 8), strides = (8, 8), and the activation function = softmax function. The loss function was categorical cross entropy. The number of intermediate layers in the FCNs can be seen in the following section.

### 3.4. Modeling

**Figure 7:**
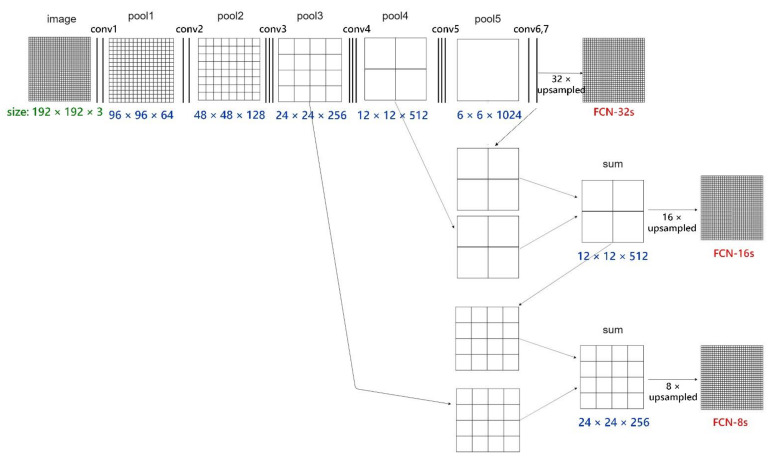
Architecture of GRI_FCN-based FCN-32s, FCN-16s, and FCN-8s and the size information of input images and feature maps in each conv-pool stage. (these FCN-type architectures contain the processes of conv1–conv7 and pool1–pool5; the architecture was referred to [37] and modified for modeling the model cases in the work).

Two types of neural network models (i.e., GRI_FCN and GRI-RRI_MCNN models) were established to examine the suitable network structures and image size. First, this study evaluated the accuracies of the FCN-32s, FCN-16s, and FCN-8s architectures by using the GRI_FCN model. Figure 7 reveals the intermediate layers (involving convolution layers and pooling layers) in these FCNs. These FCN-type architectures contained the processes of conv1–conv7 and pool1–pool5. In the figure, FCN-32s upsampled stride 32 predictions back to pixels in a single step. Subsequently, FCN-16s combined stride 16 predictions from both the final layer and the pool4 layer, at stride 16, while retaining high-level semantic information. Finally, FCN-8s used additional predictions from pool3, at stride 8, to enhance precision. The FCN employed the upsampling method to increase the pixel accuracy of the output results. Table 3 lists the total numbers of trainable variables in the FCN-32s, FCN-16s, and FCN-8s for GRI_FCN and GRI-RRI_MCNN models.

**Figure 8:**
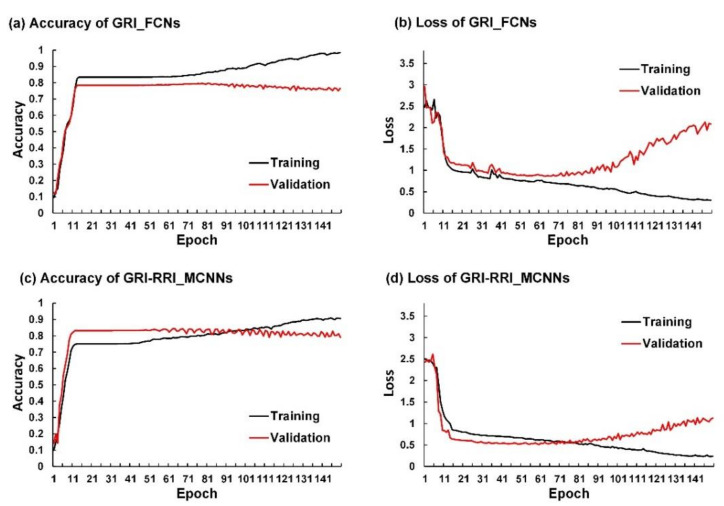
Learning curves for FCN-8s network architecture using training set (black line) and validation set (red line): (**a**) accuracy of GRI_FCNs; (**b**) loss of GRI_FCNs; (**c**) accuracy of GRI-RRI_MCNNs; (**d**) loss of GRI-RRI_MCNNs.

Figure 8 depicts the learning curves of GRI_FCNs and GRI-RRI_MCNNs for a FCN-8s network architecture using training set and validation set for a forecast horizon of 1 h. For the training set, the accuracy increased as the epoch number increased for both models (Figure 8a,c). In contrast, the accuracy for the validation set stops increasing after about 80 and 60 epochs for GRI_FCNs and GRI-RRI_MCNNs, respectively. Nonetheless, the categorical cross entropy loss decreased when the epoch number increased for both models (Figure 8b,d). In contrast, the loss values for the validation set began increasing after about 80 and 60 epochs for both models. In order to prevent overfitting, this study stopped training the models at around 80 and 60 epochs respectively for both models.

**Figure 9:**
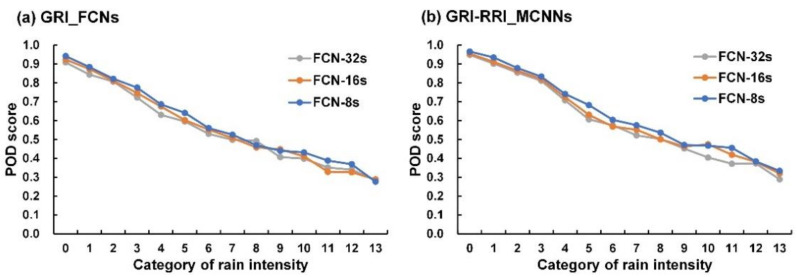
POD scores for FCN-based network architectures using validation set: (**a**) GRI_FCNs; (**b**) GRI-RRI_MCNNs.

According to [47], the probability of detection (POD) is equal to the number of hits divided by the total number of rain observations; thus it gives a measure of the proportion of rain events successfully forecast. Here, the POD measure was employed to the evaluate the accuracy of per-rain-intensity-category. Figure 9 plots the diagram for POD scores for GRI_FCNs and GRI-RRI_MCNNs as FCN-based architectures were applied. In the figure, the POD scores decreased when the rain-intensity category number increased using GRI_FCNs and GRI-RRI_MCNNs. This trend implies that these cases might correctly predict light rain but misclassify for heavier rain.

Moreover, to evaluate overall accuracy, this study adopted two commonly used categorical metrics in semantic segmentation: pixel accuracy (PA) and mean intersection over union (MIoU). The PA represents the percentage of image pixels classified correctly. The MIoU first computes the intersection over union for each semantic class and then computes the average over classes. Using the same processing, this study performed the weights training for a forecast horizon of 2–6 h. Table 4 lists the PA and MIoU performance metrics of FCN-32s, FCN-16s, and FCN-8s for forecasted horizons of 1–6 h. The results revealed that FCN-8s exhibited optimal performance in terms of the PA and MIoU. Therefore, this study used FCN-8s as the model structure.

## 4. Simulation of Typhoons

### 4.1. Accuracy Results of the Testing Set

**Figure 10:**
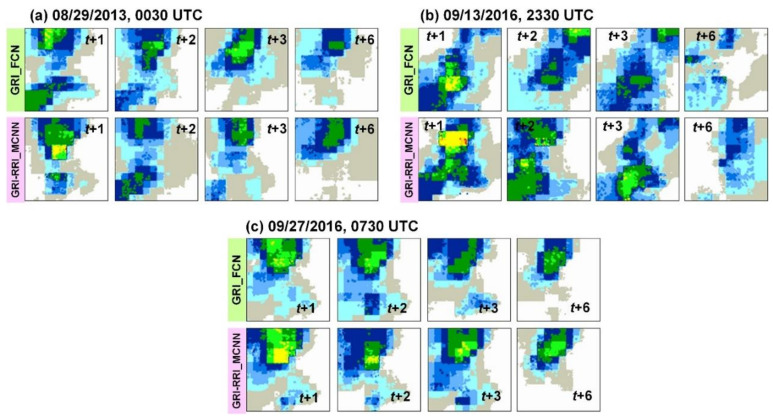
Predicted GRIs using the testing set: (**a**) Typhoons Kong-Rey, (**b**) Meranti, and (**c**) Megi.

**Figure 11:**
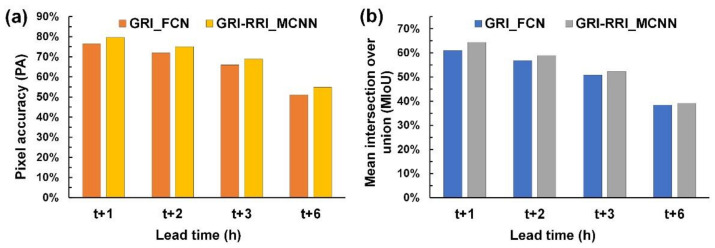
Accuracy performance of the GRI_FCN and GRI-RRI_MCNN in the testing set.

Rainfall prediction was performed for three typhoons (i.e., Kong-Rey, Meranti, and Megi) to evaluate the effectiveness of the designed GRI_FCN and GRI-RRI_MCNN models. Figure 10 displays the predicted GRI images when using the testing set. To examine the accuracy of model performance, this study also calculated the PA and MIoU metrics. Figure 11 reveals that the GRI-RRI_MCNN model outperformed the GRI_FCN model for all lead times.

### 4.2. Evaluation of Rainfall Amounts at Weather Stations

The classified outputs of every pixel in the predicted GRIs in GRI_FCN and GRI-RRI_MCNN were subsequently transformed into original rain amounts (i.e., mm/h). The research region contained 51 weather stations, comprising six CWB weather stations and 45 automatic detection stations. This study selected six CWB weather stations (i.e., Tainan, Kaohsiung, Hengchun, Dawu, Taitung, and Lanyu), which are located in various parts of southern Taiwan, to evaluate the predicted rainfall amounts.

**Figure 12:**
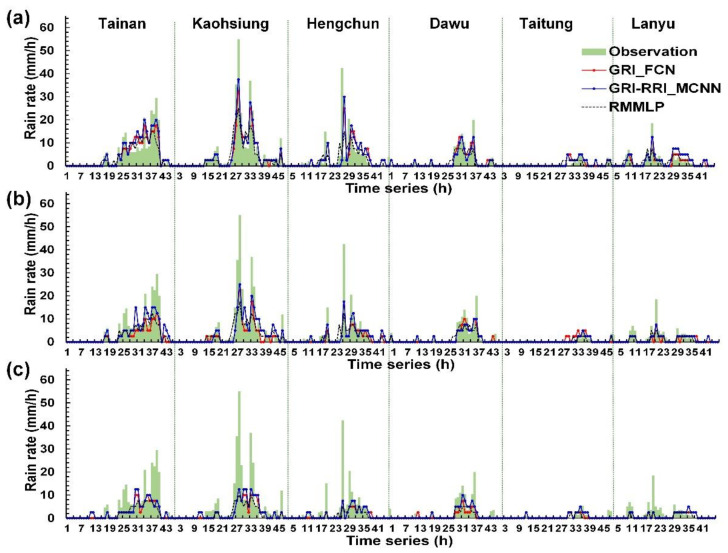
Station prediction results for Typhoon Kong-Rey at lead times of (**a**) 1 h, (**b**) 3 h, and (**c**) 6 h.

**Figure 13:**
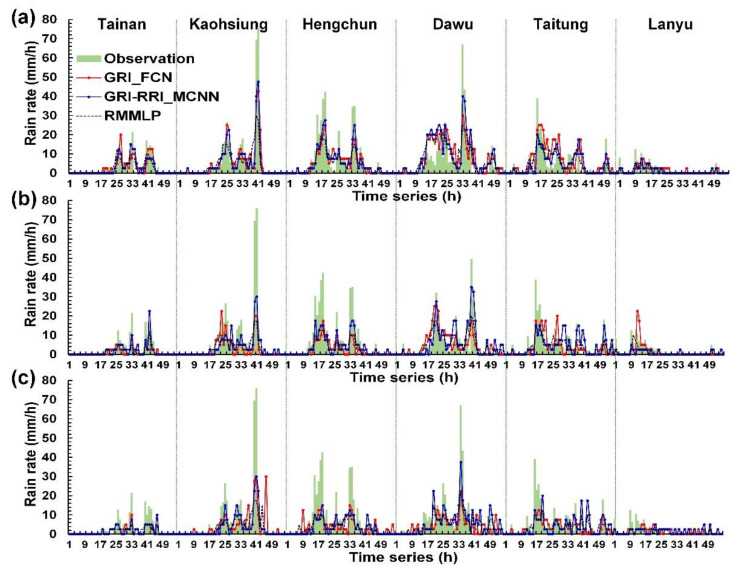
Station prediction results for Typhoon Meranti at lead times of (**a**) 1 h, (**b**) 3 h, and (**c**) 6 h.

**Figure 14:**
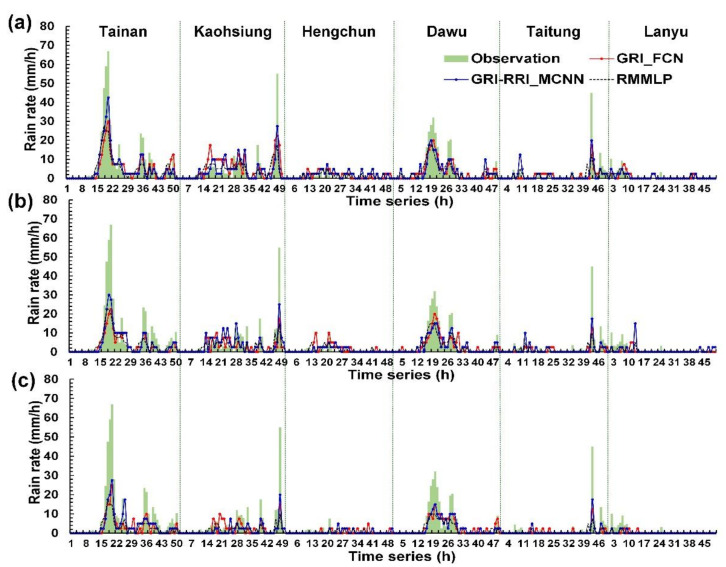
Station prediction results for Typhoon Megi at lead times of (**a**) 1 h, (**b**) 3 h, and (**c**) 6 h.

Wei and Hsieh [44] presented a radar mosaic-based multilayer perceptron (RMMLP) model, which is a conventional type of artificial neural networks that includes input, hidden, and output layers. The additional fully connected layer directly receives the cropped radar mosaic images to be flattened to a 1-D array. Here, the RMMLP model was used to a benchmark model and compared with those results made by GRI_FCN and GRI-RRI_MCNN in the six weather stations. Figure 12, Figure 13 and Figure 14 depict the rainfall prediction results of the six weather stations during Typhoons Kong-Rey, Meranti, and Megi.

**Figure 15:**
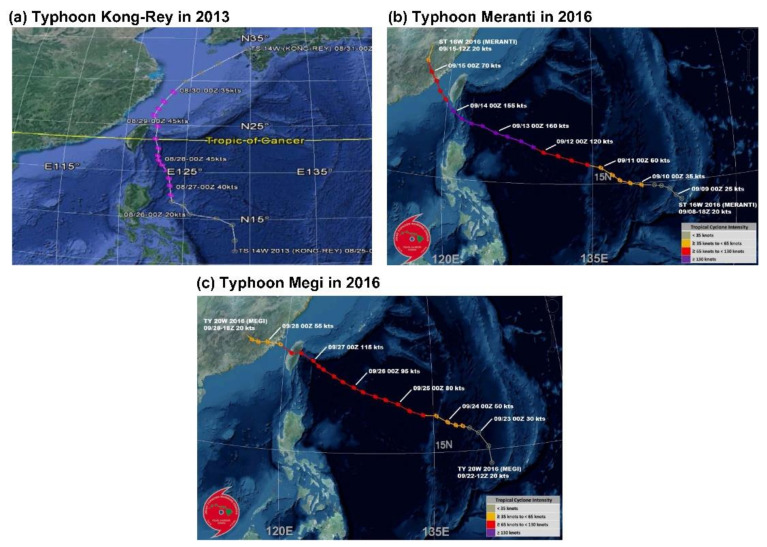
Paths of (**a**) Typhoon Kong-Rey, (**b**) Typhoon Meranti, and (**c**) Typhoon Megi (the maps were obtained from the website of the Joint Typhoon Warning Center [48].

The tracks of Typhoons Kong-Rey, Meranti, and Megi are illustrated in Figure 13. First, the center of Typhoon Kong-Rey (Figure 15a) moved northward along the eastern coast of Taiwan. Although Typhoon Kong-Rey did not land in Taiwan, its circulation caused heavy rainfall in Taiwan. The highest maximum hourly rainfall data for Typhoon Kong-Rey were observed at the Kaohsiung station (55 mm/h), followed by the Hengchun (42.5 mm/h) station. The results of the prediction models indicated that when the lead time was 1 h (Figure 12a), the trends in the predicted and observed rainfall values for the stations were consistent; however, the peak rainfall was underestimated in the prediction models. When the lead times were 3 and 6 h (Figure 12b,c), more accurate prediction results were obtained in GRI-RRI_MCNN than in GRI_FCN and RMMLP.

The center of Typhoon Meranti (Figure 15b) passed through the Bashi Channel (near the Hengchun station) and moved northwestward toward Mainland China through the Taiwan Strait. Although Typhoon Meranti did not land in Taiwan, its circulation caused heavy rainfall in Taiwan. The highest maximum hourly rainfall in the western part of the study area was observed at the Kaohsiung station (76.0 mm/h) and that in the eastern part of the study area was observed at the Dawu station (67.0 mm/h). The prediction results in Figure 13 indicate that the rainfall tendencies of each station were accurately predicted by the models. The peak rainfall and volume of underestimation increased with the prediction time.

The center of Typhoon Megi (Figure 15c) moved eastward, landed in Taiwan, and subsequently passed through central Taiwan. After landing, the typhoon circulation covered almost all of Taiwan. When the typhoon center passed through the CMR, the circulation formed a windward slope in the western side of Taiwan, which resulted in heavy rainfall in this region. The highest maximum hourly rainfall was observed at the Tainan station (67.0 mm/h), followed by the Kaohsiung station (55.0 mm/h). The prediction results in Figure 14 indicate that the models accurately predicted the rainfall trends of each station.

### 4.3. Performance Levels for Predicted Rainfall Amounts

This study employed the mean absolute error (MAE), root mean square error (RMSE), relative MAE (rMAE), relative RMSE (rRMSE), and coefficient efficiency (CE) to calculate model performance for the predicted rainfall amounts. These criteria are defined as follows:

(2) $$\mathrm{MAE}=\frac{1}{N}\sum_{t=1}^{N}\left|R_{t,\mathrm{pre}}-R_{t,\mathrm{obs}}\right|$$

(3) $$\mathrm{RMSE}=\sqrt{\frac{\sum_{t=1}^{N}{\left(R_{t,\mathrm{pre}}-R_{t,\mathrm{obs}}\right)}^{2}}{N}}$$

(4) $$\mathrm{CE}=1-\frac{\sum_{t=1}^{N}{\left(R_{t,\mathrm{obs}}-R_{t,\mathrm{pre}}\right)}^{2}}{\sum_{t=1}^{N}{\left(R_{\mathrm{obs}}-{\bar{R}}_{\mathrm{obs}}\right)}^{2}}$$

where *N* is the total number of observations, $R_{t,\mathrm{pre}}$ is the predicted rain rate at time *t*, $R_{t,\mathrm{obs}}$ is the observed rain rate at time *t*, ${\bar{R}}_{\mathrm{pre}}$ is the average of predicted rain rates, and ${\bar{R}}_{\mathrm{obs}}$ is the average of observed rain rates.

**Figure 16:**
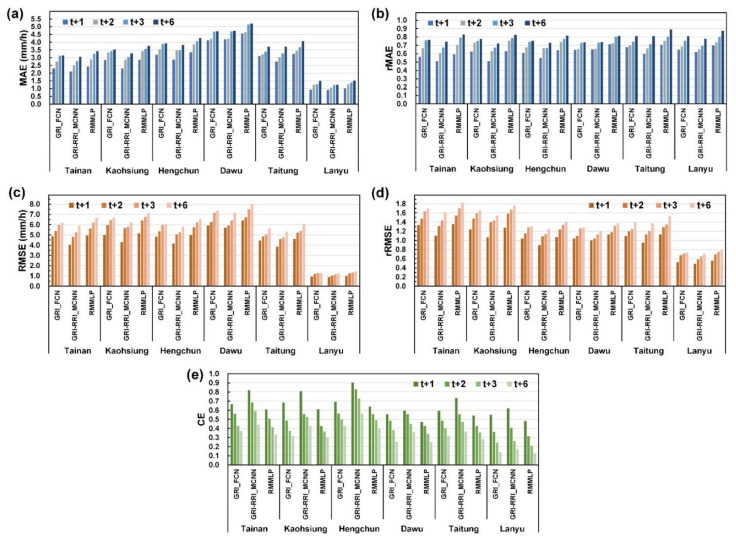
Performance levels of six stations in future (1–6 h) predictions: (**a**) MAE, (**b**) rMAE, (**c**) RMSE, (**d**) rRMSE, and (**e**) CE.

Figure 16 depicts the MAE, rMAE, RMSE, rRMSE, and CE of the results obtained at the six CWB stations. First, the absolute errors (i.e., the MAE and RMSE) were used to evaluate the obtained results (Figure 16a,c). The evaluation indicated that the absolute errors of GRI-RRI_MCNN were smaller than those of GRI_FCN and RMMLP. The values of the aforementioned parameters for the six stations in GRI-RRI_MCNN were compared. The results revealed that the Lanyu station had the lowest absolute errors among the six stations because this station was located at the sea and experienced limited rainfall and terrain effects. Among the remaining land stations, the largest absolute errors were observed at the Dawu station, followed by the Hengchun, Taitung, Kaohsiung, and Tainan stations.

Because the precipitation data of the typhoons differed among the stations, we used relative errors (i.e., the rMAE and rRMSE) to evaluate the quality of prediction. Figure 16b indicates that rMAE values of the different stations were not considerably different. Figure 16d indicates that the rRMSE exhibited greater differences among stations than the rMAE did. A comparison of the stations in mainland Taiwan revealed that the rRMSE variations at the Kaohsiung and Tainan stations were higher than those at the Dawu, Hengchun, and Taitung stations.

The overall CE was evaluated using the metric values for GRI-RRI_MCNN. As displayed in Figure 16e, the greatest CE was obtained for the Hengchun station, followed by the Tainan, Kaohsiung stations, Dawu, Taitung, and Lanyu stations. A higher prediction efficiency was obtained for the stations to the west of the CMR (i.e., the Hengchun, Tainan, and Kaohsiung stations) than for the stations to the east of the CMR (i.e., the Dawu, Taitung, and Lanyu stations).

**Figure 17:**
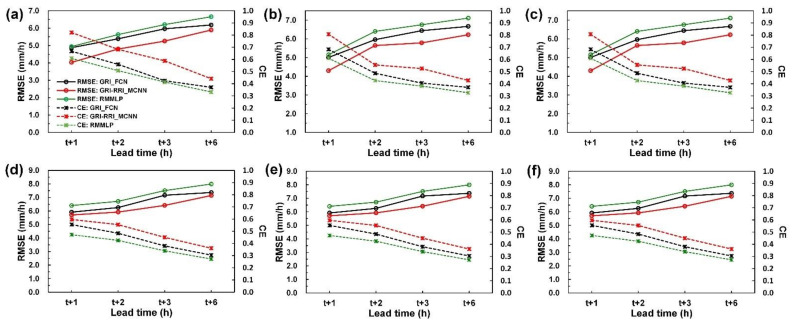
Performance levels in terms of RMSE and CE at (**a**) Tainan station, (**b**) Kaohsiung station, (**c**) Hengchun station, (**d**) Dawu station, (**e**) Taitung station, and (**f**) Lanyu station.

To determine the model performance for each station for different lead times, the RMSE and CE curves of each station were plotted (Figure 17). Figure 17a displays the RMSE–CE–lead time curves for the Tainan station. The RMSE–CE–lead time curves for the other stations are displayed in Figure 17b–f. The curves in Figure 17 indicate that the case model errors increased, and the CE gradually decreased as the prediction time increased.

To understand the improved percentage of the predictions using GRI-RRI_MCNN and GRI_FCN models compared to the benchmark (i.e., RMMLP), we defined the improvement metric IMP_C__E_, as

(5) $${\mathrm{IMP}}_{\mathrm{CE}}\;\left(\%\right)=\left({\mathrm{CE}}_{i}-{\mathrm{CE}}_{\mathrm{RMMLP}}\right)\times 100$$

where CE*_i_* is the CE value at a specific model, and CE_RMMLP_ is the CE value at the benchmark.

We calculated the average IMP_C__E_ measures of six stations for 1–6 h predictions using GRI-RRI_MCNN and GRI_FCN. After calculation, the average IMP_CE_ of GRI-RRI_MCNN and GRI_FCN were respective values of 18.9% and 6.5% for 1 h predictions, 14.9% and 5.5% for 2 h, 13.6% and 4.7% for 3 h, and 9.7% and 3.7% for 6 h. Therefore, we determined that the improvement metric resulting from GRI-RRI_MCNN was higher than that from GRI_FCN.

### 4.4. Discussion

The hyetograph error indicator performance in GRI-RRI_MCNN was superior to that in GRI_FCN and RMMLP. Better prediction of the peak rainfall time was achieved in GRI-RRI_MCNN than in GRI_FCN and RMMLP. These indicated the GRI-RRI_MCNN effectively predicted the typhoon rainfalls. However, the peak values were underestimated in these models probably because the typhoon circulation structures changed rapidly, especially under the effect of the CMR, which increased the uncertainty and difficulty in predicting transient changes in the typhoon rainfall in real time.

The movement of the typhoons affected the rainfall at each ground station. Under the effect of the CMR, if a station was windward of typhoon circulations, the rainfall was heavy; otherwise, the rainfall was relatively low. The prediction efficiency was higher for the stations to the west of the CMR (i.e., the Hengchun, Tainan, and Kaohsiung stations) than for the stations to the east of the CMR (i.e., the Dawu, Taitung, and Lanyu stations).

## 5. Conclusions

Typhoons cause severe disasters and damage in southern Taiwan. Accurate prediction of the hourly rainfall caused by typhoons can reduce life and property losses and damages. This study used the FCN model for DL image recognition to analyze the REIs and ground rain data. The collected data were analyzed for predicting the future (1–6-h) rainfall caused by typhoons in the study area. FCNs, which are extensions of CNNs, improve the defects of CNN and solve semantic segmentation problems. An FCN comprises neural net layers and performs upsampling on the feature map of the final convolution layer; thus, the FCN model can restore the size of the output results to that of the raw input images. Therefore, classification is performed for every pixel to address semantic segmentation problems.

This study collected data related to 22 typhoons that affected southern Taiwan from 2013 to 2019. Two model cases were designed. The GRI_FCN involved the use of GRIs to directly predict ground rainfall. The GRI-RRI_MCNN involved the use of REIs to retrieve the ground rainfall before the prediction of the future ground rainfall. Moreover, the RMMLP, a conventional multilayer perceptron neural networks, was used to a benchmark model. The performance of the GRI_FCN, GRI-RRI_MCNN, and RMMLP models was compared for three typhoons, namely Typhoons Kong-Rey in 2013, Meranti in 2016, and Megi in 2016. The rainfall prediction results were obtained for six ground stations in southern Taiwan (i.e., the Tainan, Kaohsiung, Hengchun, Taitung, Dawu, and Lanyu stations). This study used the GRI_FCN and GRI-RRI_MCNN models to establish a rainfall prediction model for generating the predicted GRIs of southern Taiwan. These predicted GRIs were used to assess the predicted rainfall of each station. Overall, the GRI-RRI_MCNN model enabled the typhoon rainfall in southern Taiwan to be predicted with high accuracy.

This study used the inverse distance weighting method to convert the rainfall data of ground stations into two-dimensional rainfall maps. However, the inverse distance interpolation may introduce significant artifacts such as color discrepancy and blurriness in regions where ground measurements are sparse, such as mountain area. Therefore, in the future this study suggests that remote regions could be masked in the interpolated rainfall maps where no sites are nearby and performed partial convolution [49], instead of standard convolution in the presented work.

## Figures and Tables

**Table 1:** Weather station information.

Station	Elevation (m)	Longitude (° E)	Latitude (° N)
Tainan	40.8	120.2047	22.9932
Kaohsiung	2.3	120.3157	22.5660
Hengchun	22.1	120.7463	22.0038
Dawu	8.1	120.9038	22.3557
Taitung	9.0	121.1546	22.7522
Lanyu	324.0	121.5583	22.0370

**Table 2:** Typhoon events in Taiwan from 2013 to 2019.

Typhoon	Periods	Intensity	Pressure at Typhoon Center (hPa)	Maximum Wind Speed of Typhoon Center (m/s)
Soulik	2013/07/11–13	Severe	925	51
Cimaron	2013/07/17–18	Mild	998	18
Trami	2013/08/20–22	Mild	970	30
Kong-Rey	2013/08/27–29	Mild	980	25
Usagi	2013/09/19–22	Severe	910	55
Habigis	2014/06/14–15	Mild	992	20
Matmo	2014/07/21–23	Moderate	960	38
Fung-Wong	2014/09/19–22	Mild	985	25
Noul	2015/05/10–11	Severe	925	51
Linfa	2015/07/06–09	Mild	975	30
Chanhom	2015/07/09–11	Moderate	935	48
Soudelor	2015/08/06–09	Moderate	930	48
Goni	2015/08/20–23	Severe	925	51
Dujuan	2015/09/27–29	Severe	925	51
Nepartak	2016/07/06–09	Severe	905	58
Meranti	2016/09/12–15	Severe	900	60
Megi	2016/09/25–28	Moderate	940	45
Nesat	2017/07/28–30	Moderate	955	40
Hatitang	2017/07/29–31	Mild	990	20
Hato	2017/08/20–22	Moderate	965	33
Guchol	2017/09/06–07	Mild	998	18
Bailu	2019/08/24–25	Mild	975	30

**Table 3:** Total numbers of trainable variables in the GRI_FCN and GRI-RRI_MCNN models.

Model	FCN-32s	FCN-16s	FCN-8s
GRI_FCN	1.175 × 10^8^	1.343 × 10^8^	1.351 × 10^8^
GRI-RRI_MCNN	2.350 × 10^8^	2.685 × 10^8^	2.701 × 10^8^

**Table 4:** Accuracy performance of various network structures using the validation set.

Model	Network Structures		Forecasted Horizons (h)			
			t + 1	t + 2	t + 3	t + 6
GRI_FCN	FCN-32s	PA	77.8%	73.2%	68.9%	51.2%
		MIoU	55.2%	50.3%	42.9%	30.2%
	FCN-16s	PA	78.5%	74.6%	70.8%	52.7%
		MIoU	55.1%	52.4%	47.5%	32.2%
	FCN-8s	PA	79.4%	76.9%	72.7%	56.9%
		MIoU	56.7%	53.8%	48.8%	33.5%
GRI-RRI_MCNN	FCN-32s	PA	81.9%	76.7%	71.6%	55.6%
		MIoU	57.0%	53.7%	45.6%	31.9%
	FCN-16s	PA	82.8%	77.8%	74.3%	58.5%
		MIoU	57.5%	55.2%	48.6%	34.2%
	FCN-8s	PA	83.6%	79.2%	75.3%	60.9%
		MIoU	58.7%	56.8%	50.3%	36.5%

## Data Availability

The typhoon information and radar reflectivity image were obtained from the Central Weather Bureau of Taiwan, which are available at https://rdc28.cwb.gov.tw/ (accessed on 10 January 2021) and https://e-service.cwb.gov.tw/HistoryDataQuery/index.jsp (accessed on 10 January 2021).
